# Extensive coronary artery thrombosis in a paediatric patient with Kawasaki disease: a case report

**DOI:** 10.1093/ehjcr/ytae250

**Published:** 2024-05-24

**Authors:** Tilbe Kasap, Inga Voges, Katy Rinne, Patrick Langguth

**Affiliations:** Department of Congenital Heart Disease and Pediatric Cardiology, University Hospital Schleswig-Holstein, Arnold-Heller-Str. 3, 24105 Kiel, Germany; Department of Cardiovascular Surgery, University Hospital Schleswig-Holstein, Arnold-Heller-Str. 3, 24105 Kiel, Germany; Department of Cardiovascular Surgery, University Hospital Schleswig-Holstein, Arnold-Heller-Str. 3, 24105 Kiel, Germany; Department of Congenital Heart Disease and Pediatric Cardiology, University Hospital Schleswig-Holstein, Arnold-Heller-Str. 3, 24105 Kiel, Germany

**Keywords:** Case report, Kawasaki disease, Coronary artery aneurysms, Coronary artery thrombosis, Antiplatelet and anticoagulant drugs, Cardiovascular MRI, Angiography

## Abstract

**Background:**

Kawasaki disease (KD) is a paediatric multi-system vasculitis. Mainly, the coronary arteries become affected due to acute inflammation and formation of coronary artery aneurysms (CAAs) may occur. As the size of the CAA increases, so does the risk of clinical complications and serious cardiac outcomes. These patients may experience life-threatening thrombotic coronary artery occlusion and myocardial ischaemia unless antiplatelet and anticoagulation therapy is not initiated in a timely manner.^1^

**Case summary:**

This case report presents a 12-year-old patient with KD who developed CAAs in two coronary arteries despite initial administration of intravenous immunoglobulins and acetylsalicylic acid, followed by extensive thrombosis of both coronary arteries, although antithrombotic therapy was started after the diagnosis of CAAs.

**Discussion:**

Our case is notable because of the severity of the clinical manifestation despite the administration of antiplatelet agents and anticoagulants. It could be speculated that the development of coronary thrombosis in this case might be strongly correlated with the late initiation of oral anticoagulation. The high-quality images of the affected coronary arteries in such a young patient could be of educational value.

**Learning points**

- Kawasaki disease is one of the main causes of coronary artery diseases in children.
- Current knowledge of the disease suggests that the most effective therapy for preventing thrombosis after the diagnosis of coronary aneurysms is the use of anticoagulants and antiplatelets, and late initiation or interruption of these medications may increase the risk of coronary thrombosis.

## Introduction

Kawasaki disease (KD) is a multi-systemic vasculitis of unknown aetiology that mainly affects children under 5 years of age. Kawasaki disease was first described by Tomi-saku Kawasaki in 1967 and referred to as ‘mucocutaneous lymph node syndrome’.^2^ Experience over the past 60 years has shown that KD patients are at risk for developing coronary artery aneurysms (CAA).^2^ Treatment should be initiated immediately after diagnosis and include administration of intravenous immunoglobulin (IVIG) and high-dose aspirin, which greatly reduces the incidence of CAAs.^3^ Approximately 25% of untreated patients develop CAA, compared with 4–16% with timely treatment with IVIG.^4^

Regarding aneurysm formation, coronary artery (CA) involvement can range from transient mild dilatation or ectasia to giant CAA. The average diameter of a CA is assigned a *Z*-score of 0. Positive *Z*-scores reflect larger diameters, while negative *Z*-scores reflect smaller diameters. Most individuals (∼95%) have *Z*-scores between −2 and +2, and are considered to have normal CAs. A *Z*-score between +2.0 and <+2.5 (i.e. 2–<2.5 SD above the average normalized for body surface area) is considered dilated CA. Coronary artery aneurysms are considered small if *Z*-scores are ≥2.5–<5, medium if *Z*-scores are ≥5–<10, and large or giant if *Z*-scores are either ≥10 or >8 mm in diameter. Patients with large or giant CAAs are at particular risk for cardiac events, including thrombosis or stenosis of CAs, myocardial infarction, arrhythmias, and death.^3^

Haemodynamic changes in blood flow combined with endothelial dysfunction of CAs can lead to formation of intraluminal wall-associated thrombi. Depending on the classification, prophylactic anticoagulation and/or antiplatelet therapy may be indicated. If partial thrombotic occlusion occurs, close monitoring with additional imaging is warranted. In rare cases, patients develop life-threatening thrombotic occlusion requiring coronary artery bypass grafting (CABG).^1^

We present a unique case of a 12-year-old boy with giant KD-associated CAAs who suffered extensive thrombosis and occlusion of CAs after the acute phase despite prophylactic treatment.

## Summary figure

**Figure:**
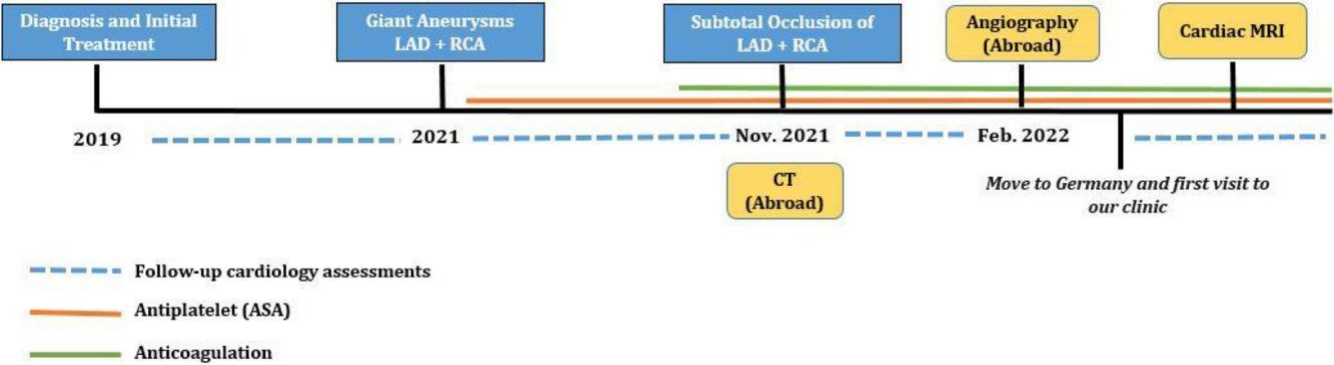


## Case presentation

**Figure 1:**
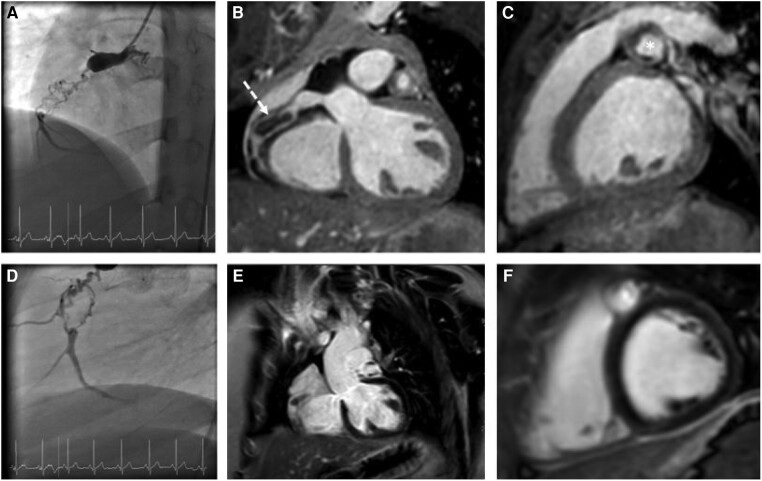
Angiography and CMR images. Angiography and CMR imaging—(*A*, *B*, *D*) chronic occlusion of the RCA with bridging collateral vessels (*B*, dotted arrow), (*C*) mural thrombi in LAD (star), (*E*, *F*) late-gadolinium enhancement without evidence for myocardial infarction.

**Figure 2:**
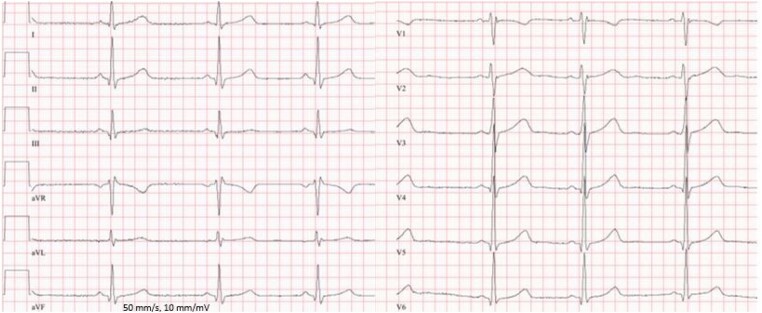
Electrocardiogram showing mild right ventricular conduction delay.

A 12-year-old boy with KD and major acquired coronary abnormalities was admitted to our hospital for follow-up at the request of his former physician in Ukraine. Initial diagnosis of KD was in 2019. He was initially treated with immunoglobulins and acetylsalicylic acid (ASA). Despite the treatment, follow-up cardiology assessments revealed giant CAAs, whereupon antiplatelet therapy and oral anticoagulation with warfarin were started. Computed tomography performed in November 2021 (images are not available), and cardiac catheterization in February 2022 showed chronic occlusion of the right coronary artery (RCA) with bridging collateral vessels (*Figure 1A* and *D*). In 2022, the patient and his family fled Ukraine to Germany because of the war. At our initial encounter, the patient was asymptomatic and clinical examination was unremarkable except for mild right ventricular conduction delay (*Figure 2*). Cardiopulmonary exercise testing showed reduced functional capacity but no ECG changes suspicious for myocardial ischaemia. Blood test showed an International Normalized Ratio (INR) value of 2.8, which lays within an effective therapeutic range (2.0–3.0). Additional cardiovascular magnetic resonance (CMR) imaging was performed and confirmed RCA and left anterior descending artery (LAD) aneurysms with a maximum transverse diameter of 11 × 12 mm (RCA) and of 11 × 15 mm (LAD). In addition, there was also evidence of chronic occlusion of the RCA with bridging collateral vessels (*Figure 1B*) and mural thrombi in LAD (*Figure 1C*; also see Supplementary material). Late-gadolinium enhancement imaging did not show any evidence for myocardial infarction suggesting a chronic aetiology of coronary artery disease and not an acute thrombotic event (*Figure 1E* and *F*). No obvious perfusion defect was detected during stress perfusion imaging. Physical stress echocardiography did not show significant wall motion abnormalities. Further history revealed that he is on antiplatelet medication since spring 2021 and that oral anticoagulation was started in October 2021. Thus, we suspect that inadequate antithrombotic medication contributed to CA thromboses. The patient’s findings were discussed in a multidisciplinary team meeting. As there was no clear evidence for myocardial ischaemia, a decision against CABG and for continuing warfarin and ASA was made.

Follow-up assessments are performed in accordance with the American Heart Association consensus statement (3). Currently, the patient is seen in the paediatric cardiology clinic every 3–6 months with investigations for inducible myocardial ischaemia every 6–12 months. Follow-up CMR was performed 5 months after the first study including stress perfusion and did not differ compared to the first scan. Clinically, the patient is well and free of cardiac symptoms.

## Discussion and conclusions

Kawasaki disease is usually a self-limiting vasculitis, although CAA may occur in ∼25–30% of untreated patients. This complication represents the most important prognostic factor and is the most common cause of death in acquired heart disease in childhood. Timely diagnosis and early initiation of treatment are important to prevent severe cardiac complications.^5^ Recent evidence shows that especially giant CAAs (especially with a *Z*-score ≥ 20) are associated with clinical complications and serious adverse cardiac events due to luminal narrowing, obstructive CA thrombosis, or life-threatening arrhythmias due to ischaemia. Therefore, not only the correct classification of CAA size in the acute phase but also long-term cardiovascular follow-up is of great importance.^1,4^

Our patient’s clinical evolution seems to show that an early diagnosis and a timely starting of IVIG therapy were ineffective in preventing CAA. According to standard therapy, 80–90% of treated patients show a clinical and biochemical remission; in the remaining percentage of patients, a persistent fever represents a sign of unresponsiveness to IVIG that is the major risk factor for the development of CA lesions.^6^

In addition, the patient developed thrombosis of RCA and LAD within the same year after the diagnosis of CAAs of both arteries, despite taking antiplatelet agents and anticoagulants. Further history revealed that the patient had been taking antiplatelet drugs since the spring of 2021, and oral anticoagulation was started in October 2021. We therefore hypothesize that interruption of antiplatelet agents after the initial diagnosis of KD and late initiation of anticoagulation may have contributed to the coronary thrombosis.

## Conclusion

Our case is notable because of the severity of clinical presentation with an early development of CAAs and extensive thrombosis with representative radiological images.

## Supplementary Material

ytae250_Supplementary_Data

## Data Availability

The data underlying this article are available in the article and in its online supplementary material.

## Lead author biography

Resident in Radiology and Neuroradiology Department, University Hospital Schleswig-Holstein, Campus Kiel, Germany.

## Supplementary material

**Consent:** Written informed consent in accordance with COPE guidelines was obtained from the parent of the patient for publication of this case report and accompanying images. A copy of the written consent is available for review by the Editor-in-Chief of this journal.

**Funding:** The authors state that this work has not received any funding.

## References

[1] van Stijn D , Schoenmaker NJ, Planken RN, Koolbergen DR, Gouw SC, Kuijpers TW, et al Myocardial infarction due to thrombotic occlusion despite anticoagulation in Kawasaki disease—a case report. BMC Pediatr 2022;22:85. 35151308 10.1186/s12887-022-03151-2 PMC8840548

[2] Kawasaki T . Acute febrile mucocutaneous syndrome with lymphoid involvement with specific desquamation of the fingers and toes in children [in Japanese]. Arerugi 1967;16:178. 6062087

[3] Newburger JW , Takahaski M, Burns JC. Kawasaki disease. J Am Coll Cardiol 2016;67:1738–1749. PMID: 27056781. 27056781 10.1016/j.jacc.2015.12.073

[4] Crindle BW , Rowley AH, Newburger JW, Burns JC, Bolger AF, Gewitz M, et al Diagnosis, treatment, and long-term management of Kawasaki disease: a scientific statement for health professionals from the American Heart Association. Circulation 2017;135:e927–e999. 28356445 10.1161/CIR.0000000000000484

[5] Leonardi S , Barone P, Gravina G, Parisi GF, Di Stefano V, Sciacca P, et al Kawasaki disease in a 3-month-old patient: a case report. BMC Res Notes 2013;6:500.PMID: 24294914; PMCID: PMC4222112. 24294914 10.1186/1756-0500-6-500 PMC4222112

[6] Kobayashi T , Inoue Y, Takeuchi K, Okada Y, Tamura K, Tomomasa T, et al Prediction of intravenous immunoglobulin unresponsiveness in patients with Kawasaki disease. Circulation 2006;113:2606–2612. 16735679 10.1161/CIRCULATIONAHA.105.592865

